# Micro 4D Imaging Sensor Using Snapshot Narrowband Imaging Method

**DOI:** 10.3390/mi14091689

**Published:** 2023-08-29

**Authors:** Wei Jiang, Dingrong Yi, Caihong Huang, Qing Yu, Linghua Kong

**Affiliations:** 1 College of Mechanical Engineering and Automation, Huaqiao University, Xiamen 361021, China; jiangwchn@foxmail.com (W.J.); nchou@hqu.edu.cn (C.H.); yuqing@hqu.edu.cn (Q.Y.); 2 School of Mechanical and Automotive Engineering, Fujian University of Technology, Fuzhou 350118, China; klh@fjut.edu.cn

**Keywords:** microarrayed spectral filter mosaic, spectral and depth imaging, snapshot imaging, micro-imaging sensor, depth from defocus

## Abstract

The spectral and depth (SAD) imaging method plays an important role in the field of computer vision. However, accurate depth estimation and spectral image capture from a single image without increasing the volume of the imaging sensor is still an unresolved problem. Our research finds that a snapshot narrow band imaging (SNBI) method can discern wavelength-dependent spectral aberration and simultaneously capture spectral-aberration defocused images for quantitative depth estimation. First, a micro 4D imaging (M4DI) sensor is proposed by integrating a mono-chromatic imaging sensor with a miniaturized narrow-band microarrayed spectral filter mosaic. The appearance and volume of the M4DI sensor are the same as the integrated mono-chromatic imaging sensor. A simple remapping algorithm was developed to separate the raw image into four narrow spectral band images. Then, a depth estimation algorithm is developed to generate 3D data with a dense depth map at every exposure of the M4DI sensor. Compared with existing SAD imaging method, the M4DI sensor has the advantages of simple implementation, low computational burden, and low cost. A proof-of-principle M4DI sensor was applied to sense the depth of objects and to track a tiny targets trajectory. The relative error in the three-dimensional positioning is less than 7% for objects within 1.1 to 2.8 m.

## 1. Introduction

Spectral imaging sensors have the ability to obtain spectral information with two-dimensional spatial information (*x*, *y*, *λ*), and they have been widely used in remote sensing [1], biomedical engineering [2,3] and food/crop quality detection [4,5]. In parallel, three-dimensional (3D) imaging, with the ability to obtain three-dimensional spatial information (*x*, *y*, *z*), plays an important role in the field of computer vision, such as in trajectory tracking [6,7], 3D reconstruction [8,9,10], and automatic driving [11,12]. In recent years, a spectral and depth (SAD) imaging method combining 3D spatial imaging and spectral imaging has been developed.

The simplest SAD imaging method is to fuse data from multiple sensors [13,14,15] to obtain 4D information (*x*, *y*, *z*, *λ*), but these methods are bulky and suffer from alignment errors. The monocular SAD imaging method can obtain 4D information (*x*, *y*, *z*, *λ*) from one imaging device, but most of them rely on scanning [16,17] or multiple frames [18,19,20,21], which leads to a low temporal resolution and requires the scene to be static. With the development of imaging technology, SAD imaging methods are gradually developing toward both monocular and snapshot [22,23,24,25]. On the one hand, the existing methods use dispersive elements [22,24,25] or a Wollaston prism [23] to map the spectrum with pixel positions, which increases the volume of the system. Most of them use computational imaging methods [22,23,24,25], which cannot display 4D data cubes in real time due to the massive computational requirements for reconstructing spectral images. On the other hand, the existing methods use light field imaging to obtain depth, which also requires increasing the volume of the imaging system. Therefore, a monocular snapshot SAD imaging method with simple implementation and low computational complexity is of great research value.

The key problem of the SAD imaging method is how to use a single two-dimensional imaging sensor to obtain multidimensional information (*x*, *y*, *z*, *λ*) in real time. Regarding snapshot spectral imaging, the snapshot narrow-band imaging (SNBI) method developed by our team [26] can capture a multispectral image in a single shot. The SNBI method uses a miniaturized narrow-band microarrayed spectral filter mosaic to transform grayscale cameras into snapshot multispectral cameras without increasing the volume of the imaging system. Regarding depth imaging, extensive research has been undertaken into depth sensing in 3D unstructured scenes using various 3D imaging methods, including light field imaging [7,22,23,24,25], multicolor depth from defocus (DFD) [27,28,29,30,31], time-of-flight [19,21,32], and multicamera stereo vision [33,34]. Most of them could not ensure that the depth obtained in a single frame and range expanded without increasing the volume of the imaging sensor. In particular, the DFD approach has unique advantages: it recovers depth by analyzing the amount of defocus blur of a single image and requires a simpler optical design. In addition, the DFD approach is a passive depth estimation method, which is not disturbed by the infrared illumination of the sun. Therefore, it can be used indoors as well as outdoors. The focus of our research is to estimate the depth from the multispectral image by using a micro-imaging sensor. We previously proposed that it was feasible to use multispectral images to detect spectral-aberration-caused defocus [35].

This study proposes a micro 4D imaging (M4DI) sensor that can dynamically capture spectral and spatial 3D information. The M4DI sensor is integrated by a mono-chromatic imaging sensor and a miniaturized narrow-band microarrayed spectral filter mosaic. The M4DI sensor has the same volume as the integrated mono-chromatic imaging sensor, and has the advantages of compactness, a light weight, and low cost. Four-channel multispectral images are obtained by a simple remapping algorithm in a single exposure. Then, we propose a method of using defocus cues from multispectral images to estimate the depth. Postprocessing requires less computational burden, which makes it possible to be applied to a real-time micro-imaging sensor. Finally, the system parameters are determined and the depth estimation performance of the prototype is tested.

## 2. System and Methods

### 2.1. Micro 4D Imaging Sensor

**Figure 1:**
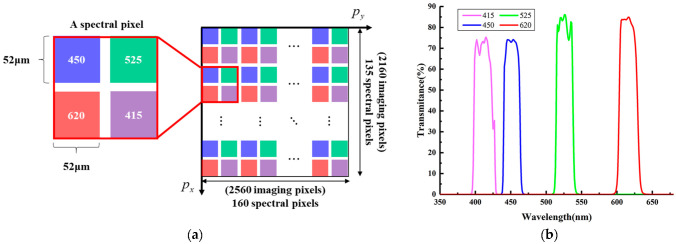
Illustration of (**a**) the geometric arrangement and (**b**) the spectral transmittance of the filter mosaic used in this study.

The M4DI sensor was developed by integrating a mono-chromatic imaging sensor with a miniaturized narrow-band microarrayed spectral filter mosaic. The filter mosaic contains 135 × 160 square compound pixels (CP), and each CP covers 16-by-16 pixels of the underneath mono-chromatic imaging sensor. A higher spatial and spectral resolution can be realized in the future by improving the manufacturing accuracy of the filter mosaic. Each CP consists of four optical microfilters arranged side-by-side in a two-dimensional manner (Figure 1a). The side length of a CP is 104 µm; hence, the side length of each microfilter is 52 µm, allowing optical light within one narrow spectral band to pass through. Jointly, a CP allows four narrow spectral bands, *B*_1_ = 450 ± 10, *B*_2_ = 525 ± 10, *B*_3_ = 620 ± 10, and *B*_4_ = 415 ± 10 nm to pass through while blocking all other wavelength light rays. The transmittance rates of all four passing bands are over 70%, which is at least four orders of magnitude higher than those of all stopping bands, which have transmittance rates lower than 0.004% (Figure 1b). Therefore, the M4DI sensor eliminates any noticeable cross-talk between different spectral bands.

**Figure 2:**
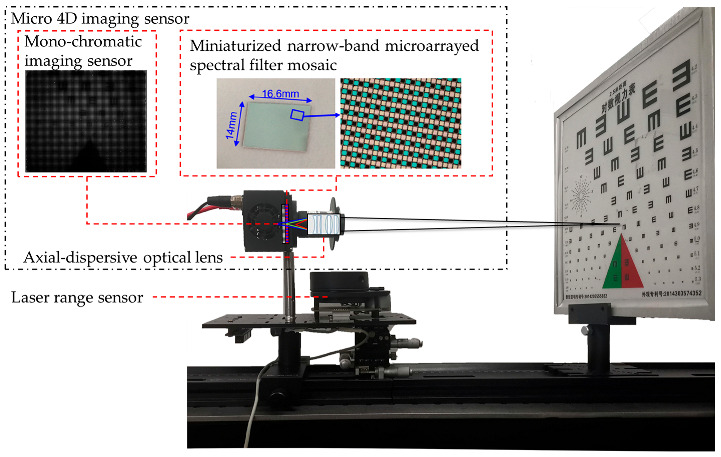
The experimental setup for the proposed M4DI sensor.

As shown in Figure 2, an experimental platform for the M4DI sensor is established, and Table 1 lists the components used in the experimental platform. The characteristic of the axial dispersion lens is that each wavelength of light has a different focal length, which enhances the defocus difference between spectral images. Light is filtered by a miniature narrow-band filter and captured by a grayscale camera. The laser range sensor is used to provide the true value of depth and is fixed with the M4DI sensor. The relative depth error of the laser range sensor is less than 0.2% in the range of 5 m.

### 2.2. Multispectral Images and Depth Map

#### 2.2.1. Multispectral Image Acquisition

**Figure 3:**
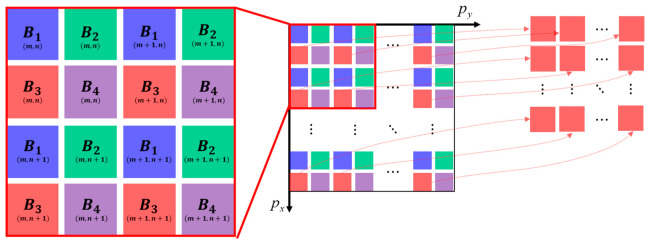
Spatial arrangement of four neighboring pixels of four bands.

Figure 3 illustrates the spatial arrangement of four neighboring pixels of multiple bands *B_i_* (*i* = 1, 2, 3, 4) within the raw image, which is determined by the filter mosaic shown in Figure 1a. At a single exposure, the M4DI sensor captured a raw image of the scene (Figure 3). A simple remapping algorithm was developed to separate the raw image *R*(*r*,*c*) (*r* = 1, 2, …, 2560; *c* = 1, 2, …, 2160) into four narrow spectral band images *B_i_* (*m*,*n*), where *i* = 1, 2, 3, 4 and *m* = 1, 2, …, 135; *n* = 1, 2, …, 160, according to Equation (1):

(1) $$B_{i}\left(m,n\right)=\frac{\sum_{x=2}^{5}\sum_{y=2}^{5}R\left(16m+x+8\times mod\left(i-1,2\right),16n+y+8\times \left\lfloor \frac{i-1}{2}\right\rfloor \right)}{16},$$

where *mod*(*a*,*b*) is the remainder of the division of a and b. The symbol ⌊ ⌋ is rounded toward negative infinity. In a spectral pixel, all the gray values in the coverage area of an *i* channel filter are averaged. Due to the limitation of the manufacturing technology, there is a gap between different spectral bands, so a 4 × 4 area is selected as the window of each spectrum (*x*, *y* = 2, 3, 4, 5).

#### 2.2.2. Depth from Multispectral Imaging with Chromatic Aberration

The key of DFD is how to obtain defocus information from the blurred image. A blurred image can be modeled as a convolution of a clear image with a point spread function (PSF). For circular apertures, the defocus pattern can be approximated by the Gaussian function $g\left(x,\sigma \right)=1/\sqrt{2\pi \sigma^{2}}\times exp\left(-x^{2}/2\sigma^{2}\right)$. The standard deviation *σ* of the Gaussian function represents the degree of blur. The defocused image *I*(*x*) captured by the imaging sensor can be represented by the following formula:

(2) $$I\left(x\right)=f\left(x\right)⊗g\left(x,\sigma \right)+n\left(x\right),$$

where *x* is the pixel coordinate of the image, ⊗ is a convolution symbol, and *n*(*x*) is random noise.

The chromatic aberration of the lens is characterized by each wavelength of light having a different focal length, which is caused by the different transmittance of each wavelength of light. In general, chromatic aberration is eliminated in the postprocessing step. Our research is focused on estimating depth from blur differences in multispectral images. The relationship between defocus cues *σ_i_* of the *i*th spectral channel *λ_i_* and depth *d* is as follows:

(3) $$\sigma_{i}\left(x\right)=kDs\left|\frac{1}{d_{i}}-\frac{1}{d}\right|,$$

where *D* is the optical aperture; *k* is a constant related to the imaging system; *s* is the distance between the sensor and the lens; and *d_i_* is the optimal focus distance (OFD) corresponding to the *i*th spectral channel *λ_i_*.

Generally, defocus cues are easily estimated at the edge of the image texture [36]. The step blur edge is the main type in the image texture. Calculating the defocus degree of the step edge can be used to estimate the depth. The image at the edge can be expressed as $f\left(x\right)=\left[Au\left(x\right)+B\right]⊗g\left(x,\sigma_{0}\right)$. The function *u*(*x*) is the step function, and *σ*_0_ is a fixed standard deviation of the step blur edge. *A* is the amplitude, and *B* is the offset. The defocused image captured by the imaging sensor can be represented by the following formula:

(4) $$I_{i}\left(x\right)=\left[Au\left(x\right)+B\right]⊗g\left(x,\sigma_{0}\right)⊗g\left(x,\sigma_{i}\right)+n\left(x\right),$$

where *I_i_*(*x*) is the pixel value at the image position *x* of the *i*th spectral channel *λ_i_* and $\sigma_{i}$ is the standard deviation of the *i*th spectral channel *λ_i_.* Median filtering is applied to the multispectral image captured by the M4DI sensor to eliminate noise. Before depth estimation, one preprocessing procedure was applied to normalize the four spectral images to make their amplitude *A* comparable. The normalized spectral image *I*(*λ*) is obtained using Equation (5):

(5) $$I\left(\lambda \right)=\frac{B\left(\lambda \right)-B_{min}\left(\lambda \right)}{B_{max}\left(\lambda \right)-B_{min}\left(\lambda \right)}.$$

where *B*(*λ*) is the spectral image obtained using Equation (1), *B_min_*(*λ*) is the minimum gray value of the spectral image, and *B_max_*(*λ*) is the maximum gray value of the spectral image.

Then, the gradient of the blurred image becomes:

(6) $$\nabla_{i}\left(x\right)=\frac{A}{\sqrt{2\pi \left(\sigma_{0}^{2}+\sigma_{i}^{2}\right)}}exp\left(-\frac{x^{2}}{2\left(\sigma_{0}^{2}+\sigma_{i}^{2}\right)}\right).$$

Write $\nabla_{i}\left(0\right)$ as $\nabla_{i}$. At the edges (*x* = 0), the gradient becomes:

(7) $$\nabla_{i}=\frac{A}{\sqrt{2\pi \left(\sigma_{0}^{2}+\sigma_{i}^{2}\right)}}.$$

According to Equation (7), the edge gradient of any three wavelength images is used to eliminate the fixed standard deviation *σ*_0_. For convenience, the following equation is denoted as *M*:

(8) $$M=\frac{\frac{1}{\nabla_{i}^{2}}-\frac{1}{\nabla_{j}^{2}}}{\frac{1}{\nabla_{k}^{2}}-\frac{1}{\nabla_{j}^{2}}}=\frac{\sigma_{i}^{2}-\sigma_{j}^{2}}{\sigma_{k}^{2}-\sigma_{j}^{2}}\;\;i\neq j\neq k.$$

Substituting Equation (3) into Equation (8) and defining $D_{i}=\frac{1}{d_{i}}$:

(9) $$\hat{d}\left(\nabla_{i},\nabla_{j},\nabla_{k}\right)=2\times \frac{\left[M\times \left(D_{k}-D_{j}\right)-\left(D_{i}-D_{j}\right)\right]}{\left[M\times \left(D_{k}^{2}-D_{j}^{2}\right)-\left(D_{i}^{2}-D_{j}^{2}\right)\right]}\;\;i\neq j\neq k.$$

It can be seen from Equation (9) that the depth can be estimated from three spectral images. In the imaging system, there is a limit to the gradient change perceived by the image sensor. When the blur size is too large, it may cause a gradient change that is too small to be sensed by the sensor. Therefore, three spectral images with the closest central wavelengths are applied to Equation (9), and other spectral images provide depth estimation in a new range. In this paper, four spectral images are used for depth estimation:

(10) $$\hat{d}=\left\{\begin{array}{cc} \hat{d}\left(\nabla_{1},\nabla_{2},\nabla_{3}\right) & if\;\nabla_{2}\;or\;\nabla_{3}=max\left(\nabla_{1},\nabla_{2},\nabla_{3},\nabla_{4}\right)\\ \hat{d}\left(\nabla_{1},\nabla_{2},\nabla_{4}\right) & if\;\nabla_{1}\;or\;\nabla_{4}=max\left(\nabla_{1},\nabla_{2},\nabla_{3},\nabla_{4}\right)\;\\ 0 & else \end{array}\right..$$

The sparse depth is obtained by Equation (10) and the depth value is only estimated at the edge. A dense depth map, wherein each pixel within the image has a depth value, can be obtained from the sparse depth map using existing algorithms [37] with some modifications. We use the matting Laplacian method to interpolate the sparse depth $\hat{d}$ into a dense depth map *δ*. The depth map interpolation method needs to minimize the following cost function:

(11) $$E\left(\delta \right)=\delta^{T}L\delta +\rho {\left(\delta -\hat{d}\right)}^{T}H\left(\delta -\hat{d}\right),$$

where *L* is the Laplace matrix, which was proposed in [37]; *ρ* is a smoothing constant; *H* is a diagonal matrix whose element *H_mm_* is equal to 1 if the inequality $\hat{d}\left(m\right)\neq 0$ is satisfied at pixel *m*. Equation (11) is derived and the derivative is made 0:

(12) $$\left(L+\rho H\right)\delta =\rho H\hat{d},$$

Equation (12) can be rewritten in the following way:

(13) $$\delta ={\left(L+\rho H\right)}^{-1}\rho H\hat{d}.$$

The value of *ρ* is determined by the camera system or estimation method. In our system, we use a fixed *ρ* value of 0.001.

## 3. Results and Discussion

**Figure 4:**
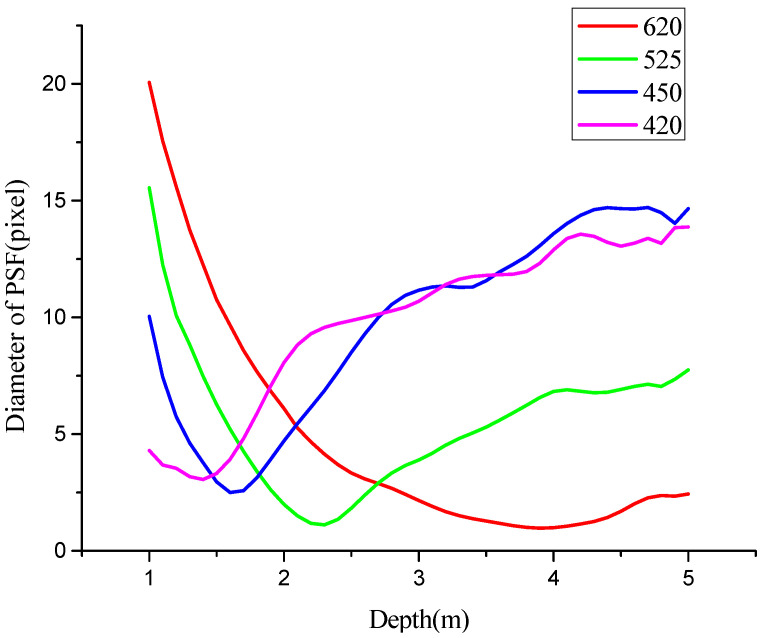
Variations in each spectral channel defocus of the dispersive optical lens.

According to Equation (9), it is necessary to determine the OFD of each spectral channel. The OFD can be obtained by illuminating the imaging sensor with a point light source. Figure 4 illustrates the amount of bandwise defocus variation with changes in depth, measured as the full-width-half-maximum (FWHM) of a point light source imaged by the M4DI sensor. The depth corresponding to the minimum value of the FWHM is the OFD. Figure 4 measures the OFD as *d*_4_ = 1.4 m, *d*_3_ = 4 m, *d*_2_ = 2.3 m, and *d*_1_ = 1.6 m. Note that this OFD is a fixed value in our system because the focal length in each channel and the lens are fixed.

**Figure 5:**
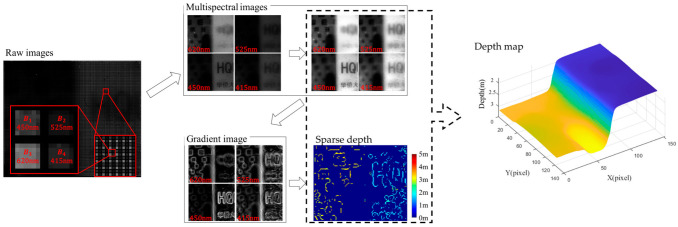
The image processing process of the M4DI sensor.

The image processing process of the M4DI sensor is shown in Figure 5. At a single exposure, four spectral images are separated from the original images. The spectral images are normalized, and then their gradient is calculated. We use the Canny edge detector to perform edge detection and estimate the depth at the edge (sparse depth). Finally, a depth map is obtained by filling the sparse depth according to the normalized spectral images.

**Figure 6:**
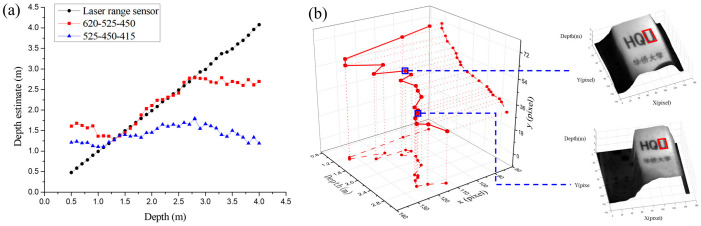
Illustration of (**a**) the result of depth estimation and (**b**) the track recovery result of the tiny character “U” (size: 10 × 17 mm).

A plane sample was placed at intervals of 10 cm in the range of 1~4 m to test the accuracy and range of depth estimation. The result of the depth estimation is shown in Figure 6a. The true depth, illustrated by the black curve, was measured using a laser range sensor, which has 1 mm accuracy for a depth range within 5 m. When three channel images of 620 nm, 525 nm, and 450 nm are used as input images, the depth estimation range is 1.3~2.8 m. When three channel images of 525 nm, 450 nm, and 415 nm are used as input images, the depth estimation range is 1.1~1.4 m. Therefore, the depth estimation range of the M4DI sensor is 1.1 to 2.8 m. The maximum relative error of the M4DI sensor is within 7%. Our team further applied the M4DI sensor to recover the trajectory of a box printed with tiny characters. The central pixel position and depth of the character “U” (size: 10 × 17 mm) are given by template matching. The trajectories sampled at 16 different positions are shown in Figure 6b. This shows that the M4DI sensor could be applied to the field of target recognition and tracking.

**Figure 7:**
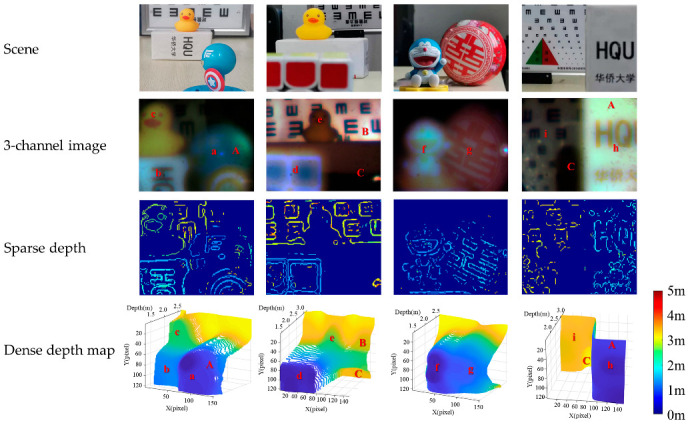
Depth estimated by the M4DI sensor: the first row shows the images of four different scenes captured by a smartphone; the second row shows four pseudocolor images composed of 620 nm, 525 nm, and 450 nm channel images; the third row shows the sparse depth (only at edges) for the four scenes; and the fourth row shows the dense depth map for the four scenes.

We further conducted a qualitative evaluation to test whether the M4DI sensor could conduct 3D sensing of a complicated object. Experimental samples were placed at different depths. The result of depth estimation is shown in Figure 7. Multiple factors could affect the depth measurement accuracy. One is the uneven reflectivity of the surface material, which would enhance the received light intensity, and hence, the gradient of a particular band if the reflectivity peaks at that band. Strong reflectivity in any one of the four narrow bands causes an apparently shallower depth than the real depth. Another is that no texture information is obtained, which means no information for depth in the passive depth estimation method. Specifically, as shown in Figure 7, the sample has no texture (symbol A), strong specular reflection (symbol B), and weak illumination (symbol C). Even with these error-inducing factors, the far–near relationship between samples can be distinguished, that is, a < b < c, d < e, f < g, and h < i.

## 4. Conclusions

In conclusion, this paper presents an M4DI sensor for multispectral depth imaging, which can obtain narrow-band spectral images of four channels, and the relative error in the depth recovery is less than 7% in the depth range of 1.1 to 2.8 m. The M4DI sensor was developed by integrating a mono-chromatic imaging sensor with a miniaturized narrow-band microarrayed spectral filter mosaic, which made it have the same volume as the integrated mono-chromatic imaging sensor. The advantages of the proposed M4DI sensor include its high efficiency of generating 4D cubic data, its extended depth of focus when combining its various spectral bands, its compactness, its light weight, and its ability to work in passive environments. These advantages make it unique among SAD imaging methods, as none of the existing SAD imaging methods can obtain a multispectral depth image in a single frame without increasing the volume of the imaging sensor. The M4DI sensor was applied to sense the depth of objects and to track tiny target trajectories. The M4DI sensor, as a miniaturized real-time imaging system, has the potential to identify and track targets in a narrow space.

## Figures and Tables

**Table 1:** List of components used in the experimental platform.

Components	Manufacturer	Function
Mono-chromatic imaging sensor	United Scientific Camera & Imaging Corp, (A55-G17M)	Capture gray image
Miniaturized narrow-band microarrayed spectral filter mosaic	Self-built	Convert gray image into narrow-band spectral image
Axial-dispersive optical lens	Self-built	Produce chromatic dispersion
Laser range sensor	Zhiwei Robotics Corp, (RPLIDAR A1M8-R6)	Obtain true depth for testing performance

## Data Availability

Data sharing is not applicable to this article.
